# Integrated Transcriptomic and Metabolomic Analysis of Starch Biosynthesis in Different Spatial Regions of Tetraploid Potato Tubers

**DOI:** 10.3390/biology15181655

**Published:** 2026-09-18

**Authors:** Huanhuan Lian, Chao Zhang, Guangji Ye, Wang Su, Shenglong Yang, Yun Zhou, Yongzhen Ma, Jian Wang

**Affiliations:** 1 Academy of Agriculture and Forestry Sciences, Qinghai University, Xining 810016, China; 18297189063@163.com (H.L.); 2025990141@qhu.edu.cn (C.Z.); 13997089595@163.com (G.Y.); ysl890224@163.com (S.Y.); zhouyun75@163.com (Y.Z.); 2025990004@qhu.edu.cn (Y.M.); 2 Qinghai Provincial Key Laboratory of Potato Breeding, Qinghai University, Xining 810016, China; 3 Ministry of Education Engineering Research Center of Potato in Northwest Region, Qinghai University, Xining 810016, China

**Keywords:** tetraploid potato, tuber spatial regions, starch biosynthesis, transcriptome, metabolome, carbon flux regulation

## Abstract

Potato tubers exhibit prominent spatial heterogeneity in starch accumulation across distinct anatomical zones, yet the underlying transcriptional and metabolic regulatory mechanisms remain unclear, especially the molecular basis for divergent starch deposition between high- and low-starch tetraploid cultivars. In this study, the high-starch cultivar Atlantic (DX) and low-starch cultivar Dingshu No. 1 (D) were used as experimental materials. Dynamic starch contents of the tuber cortex (CR), perimedullary region (PMR), and inner medullary region (IMR) were measured throughout starch biosynthetic stages. Integrated untargeted metabolomics and RNA-seq transcriptomics were performed to compare transcriptional and metabolic differentiation between CR and IMR with the largest starch gap, followed by qRT-PCR verification of hub genes. Physiological results showed that starch content in both cultivars followed the gradient CR > PMR > IMR, and inter-regional starch disparities expanded continuously with tuber development; low-starch D had lower overall starch levels and milder tissue differences than DX. Transcriptomic analysis revealed massive transcriptional activation in the cortex of both cultivars, while D displayed far more dramatic transcriptional divergence between CR and IMR than DX. Core transcription factors including AP2/ERF, bHLH and MYB were identified to mediate tissue-specific starch synthesis. Metabolomic data demonstrated severe carbon metabolic polarization in D (hyperactive cortex, weak inner medulla), whereas DX maintained balanced sugar metabolism across whole tuber tissues. Integrated multi-omics analysis of the starch and sucrose pathway (ko00500) screened 18 key structural genes. High-starch DX upregulated starch synthetic and sugar transport genes while repressing starch hydrolysis and carbon diversion genes, realizing coordinated whole-tuber carbon flux toward starch production. In contrast, D showed disrupted regulatory networks, excessive carbon shunting to secondary metabolism and aggravated starch degradation, limiting starch accumulation. Three conserved negative regulatory candidate genes, LOC102593331 (*TPS*), LOC102584887 (*AMY*), and LOC102580651 (*EN*), were characterized via tissue expression profiling: TPS dominates upstream carbon flux allocation, EN competes for starch precursors during cell wall synthesis, and AMY accelerates starch breakdown. This study systematically uncovered the molecular framework of spatial starch heterogeneity in tetraploid potato tubers, clarified the essential distinctions in carbon partitioning strategies between high- and low-starch cultivars, and provided valuable gene resources and theoretical support for high-starch potato molecular design breeding.

## Simple Summary

Spatial differences in starch deposition widely exist within potato tubers, yet the molecular regulatory mechanisms and the distinctions between high- and low-starch tetraploid cultivars remain poorly understood. We monitored dynamic starch contents across three anatomical zones of tubers in Atlantic (high-starch) and Dingshu No. 1 (low-starch), and integrated RNA-seq and untargeted metabolomics to dissect transcriptional and metabolic variations between the cortex and inner medulla. Both varieties displayed a starch gradient of cortex > perimedullary region > inner medulla, yet the low-starch cultivar presented lower total starch, milder inter-tissue disparities, and severe carbon metabolism polarization, in contrast to the balanced carbon metabolism in the high-starch cultivar. Eighteen key structural genes and three conserved inhibitory genes (TPS, AMY, EN) governing carbon flux and starch turnover were verified: the high-starch variety orchestrates holistic carbon flux toward starch biosynthesis via transcriptional modulation, while regulatory disorder, redundant carbon shunting to secondary metabolism and accelerated starch degradation collectively lead to inferior starch accumulation in the low-starch cultivar. Our findings decipher the molecular framework underlying tuber starch spatial heterogeneity and provide critical references for high-starch potato molecular breeding.

## 1. Introduction

Potato (*Solanum tuberosum* L.) is the most widely cultivated and consumed tuber food crop worldwide, occupying an irreplaceable strategic position in safeguarding national food security, optimizing dietary structures, and supporting industrial processing sectors [1,2]. Distinct from cereal crops that store assimilates within grains, potato develops underground vegetative tubers as the dominant sink organ for carbohydrate reserves, and starch accounts for the largest proportion of tuber dry weight [3]. Starch content directly determines tuber yield, taste, and cooking properties, and is also the pivotal raw material for starch manufacturing, food deep processing and pharmaceutical industries [4]. Accordingly, breeding potato varieties with high and stable starch content has long been a primary goal for potato quality improvement and industrial utilization [5]. Alongside the advancement of molecular precision breeding and rapid expansion of the starch processing industry, traditional yield-centered breeding modes can hardly satisfy industrial requirements for stable high-starch potato germplasm. Unraveling the molecular regulatory network underlying starch biosynthesis and spatial accumulation within tubers, and excavating hub functional genes and regulatory elements have become urgent research priorities to break through bottlenecks in potato quality breeding [6].

Photosynthate translocation and source–sink partitioning constitute the foundational physiological framework governing tuber starch accumulation. In the potato carbon flow system, mature functional leaves serve as carbon sources that fix carbon dioxide via photosynthesis to synthesize sucrose; sucrose is transported downward through the phloem to underground stolons and developing tubers (sink tissues), where sucrose is degraded, reconverted and ultimately assimilated into starch for storage [7]. Numerous classic physiological studies have clarified the overall carbon allocation rules during potato growth: carbon partitioning is dynamically reshaped throughout tuber initiation, expansion and maturation stages, and sink strength of tubers dominates the efficiency of photosynthate import [8]. Nevertheless, most prior source–sink studies focused on whole-plant carbon distribution between leaves and entire tubers, ignoring the redistribution and internal partitioning of imported carbon substrates among different anatomical compartments inside tubers.

Tuber morphogenesis and amyloplast differentiation lay the cytological basis for heterogeneous starch deposition. The potato tuber is structurally divided into the cortex, perimedullary region and inner medulla along the radial axis, which derive from different meristematic tissues and undergo asynchronous cell expansion and amyloplast development. Existing cytological observations have documented that cells in the perimedullary region feature larger volumes, more abundant amyloplasts and faster amyloplast proliferation rates, whereas inner medullary cells mature slowly with smaller starch grains, and cortical cells are compact with limited starch storage capacity [9]. Amyloplast proliferation, swelling and starch granule packaging directly govern starch synthesis efficiency in distinct tissues [9,10]. A body of previous work has characterized the developmental progression of amyloplasts during tuber bulking, yet few studies have linked cytological amyloplast variation to quantitative starch accumulation discrepancies across tuber zones, nor clarified how tissue anatomical properties constrain carbon utilization efficiency for starch synthesis.

Tuber development and starch accumulation represent pivotal processes shaping potato yield and quality, which involve complex biological programs including amyloplast differentiation and development, photosynthate translocation and partitioning, and synergistic regulation of multiple metabolic pathways [11,12,13]. Existing studies have confirmed that potato tubers are not homogeneous storage organs; distinct anatomical zones (cortex, perimedullary region and inner medullary region) exhibit remarkable differences in cellular morphology, tissue structure and physiological function, accompanied by evident spatial heterogeneity in starch synthesis rate, accumulation quantity and spatiotemporal dynamics. Nevertheless, most domestic and international studies on potato starch accumulation have focused on overall starch content variations among different cultivars, developmental stages or cultivation environments, or functional validation of single key starch synthase genes [6,10]. Research targeting differential starch accumulation across distinct tuber anatomical regions remains limited.

Physiologically, preliminary studies have verified uneven starch deposition in different tuber regions, yet systematic dissection of synergistic relationships among dynamic starch accumulation patterns and fresh weight partitioning across tuber zones of high- and low-starch cultivars is lacking. The intrinsic linkage between tissue structural development and spatial starch deposition also remains unclarified. At the molecular regulatory level, previous research has identified core structural genes (e.g., starch synthases, sucrose synthases) and partial transcription factors modulating starch biosynthesis [14,15]. However, most relevant analyses were performed using mixed whole-tuber samples, ignoring transcriptional and metabolic differentiation among internal anatomical regions. Such an experimental design fails to precisely distinguish tissue-specific variations from cultivar genetic disparities, making it difficult to unravel core regulatory mechanisms underlying differential starch accumulation within tubers. Furthermore, most current investigations rely on individual transcriptomic or metabolomic datasets, which cannot elucidate the complete regulatory cascade linking gene expression, metabolite accumulation and phenotypic formation. In particular, the absence of systematic integrated transcriptome–metabolome regulatory networks governing differential starch deposition across tuber zones of high- versus low-starch cultivars severely restricts the identification and application of precision breeding targets for high-starch potato varieties.

Cultivated tetraploid potato possesses a complex autotetraploid genetic background characterized by tetrasomic inheritance, and its starch regulatory network is governed by polygenic coordination and multi-metabolic pathway coupling. Single-omics approaches are insufficient to comprehensively resolve the intricate regulatory machinery of this complex quantitative agronomic trait. Integrated transcriptomic and metabolomic profiling enables systematic interpretation from transcriptional regulation to terminal metabolic phenotypes, serving as an efficient strategy to dissect molecular mechanisms of crop complex traits and mine core functional genes. This combinatorial omics strategy has been widely adopted to investigate crop quality formation, yield construction and abiotic/biotic stress tolerance in tuber and grain crops [16,17].

On this basis, the present study utilized two potato cultivars with divergent starch phenotypes, the high-starch cultivar Atlantic and the low-starch cultivar Dingshu No. 1, as experimental materials. We systematically quantified starch content and fresh weight proportion in the cortex (CR), perimedullary region (PMR) and inner medullary region (IMR) during critical starch biosynthetic stages to clarify physiological rules governing spatiotemporal starch deposition and tissue structural development. Further integrated transcriptomic and untargeted metabolomic analyses were conducted to comparatively characterize transcriptional and metabolic differentiation across tuber zones of the two cultivars. Core differentially expressed genes (DEGs), key differential metabolites (DMet) and pivotal pathways regulating tissue-specific starch accumulation were screened, followed by construction of an integrated transcriptional–metabolic regulatory network for starch biosynthesis. Quantitative real-time PCR (qRT-PCR) was performed to validate expression patterns of hub genes.

This study aims to dissect divergent starch regulatory modules between high- and low-starch potato cultivars, supplement the insufficient research on spatial starch accumulation mechanisms inside potato tubers, and excavate core candidate genes and regulatory targets applicable to precision high-starch breeding. The findings will provide systematic theoretical support and genetic resources for quality improvement, molecular design breeding and in-depth dissection of starch biosynthetic pathways in tetraploid potato, while offering critical references for research on storage substance spatial accumulation in tuberous root crops.

## 2. Materials and Methods

### 2.1. Plant Materials and Field Cultivation

The high-starch potato cultivar Atlantic and the low-starch potato cultivar Dingshu No. 1 were used as experimental materials in this study, and were provided by Qinghai Academy of Agriculture and Forestry Sciences. A unified sample coding system was defined to avoid naming confusion: the prefix DX represents the cultivar Atlantic, and D represents the cultivar Dingshu No. 1; IMR refers to tuber inner medulla, PMR refers to perimedullary region, and CR refers to tuber cortex. The formal standardized labels are defined as: DX-IMR (inner medulla of Atlantic tubers), DX-PMR (perimedullary region of Atlantic tubers), DX-CR (cortex of Atlantic tubers), D-IMR (inner medulla of Dingshu No. 1 tubers), D-PMR (perimedullary region of Dingshu No. 1 tubers), and D-CR (cortex of Dingshu No. 1 tubers).

For convenience in early plotting, temporary numerical codes were adopted in partial figures: DX-1 = DX-IMR (Atlantic inner medulla), DX-3 = DX-CR (Atlantic cortex); D-1 = D-IMR (Dingshu No.1 inner medulla), D-3 = D-CR (Dingshu No.1 cortex). All subsequent text descriptions adopt the standardized combined naming mode of cultivar prefix + tissue abbreviation; the full names “Atlantic” and “Dingshu No.1” will not be repeatedly used independently in the manuscript to avoid confusion caused by mixed use of full names and abbreviations.

Standardized field management regimes were applied during cultivation. Intertillage and weeding were conducted immediately after seedling emergence. Urea was topdressed together with hilling at the squaring stage, and potassium fertilizer was applied at the tuber initiation stage. Timely irrigation was carried out in accordance with soil moisture throughout the entire growth cycle to keep soil water content ranging from 60% to 70%. Pests and diseases such as potato late blight and aphids were managed by combined physical and biological control methods to ensure healthy growth and development of potato plants.

### 2.2. Determination of Starch Content

Fresh potato tubers harvested in the field were promptly transported back to the laboratory, rinsed with clean water to remove surface soil, and the surface moisture was blotted up with filter paper. Tubers were longitudinally sliced manually, and the cortex (CR), perimedullary region (PMR) and inner medulla region (IMR) were precisely separated according to tuber anatomical structures. Transitional tissues between different regions were completely discarded to ensure sample purity prior to starch measurement.

Starch content was measured using the plant starch extraction kit (Catalog No. BC0700) purchased from Solarbio Science & Technology Co., Ltd. (Beijing, China). The experiment was performed strictly in accordance with the kit instructions and national standard GB 5009.9-2016. Briefly, 0.1 g of fresh tuber samples were accurately weighed, mixed with 500 μL extraction solution, and homogenized by grinding under ice bath conditions. The homogenate was heated in a boiling water bath for 10 min, followed by centrifugation at 8000 rpm for 10 min. The supernatant was collected as the test solution. Buffer solution, enzyme solution, chromogenic reagent, and other reagents were sequentially added following the kit reaction system. After incubation at a constant temperature of 37 °C for 30 min, the absorbance value was detected at a wavelength of 540 nm, and the starch content was calculated based on the standard curve.

### 2.3. Analysis of Starch Content in Distinct Tuber Regions

To clarify the dynamic starch accumulation patterns in three tuber regions (inner medulla, perimedullary region, and cortex) of potato tubers, eight consecutive tuber developmental stages (S1–S8) were sampled for the cultivars Atlantic and Dingshu No. 1. Starch content was determined for the three tuber regions, with three biological replicates per sample.

A two-way repeated measures analysis of variance (ANOVA) was performed independently for each cultivar using SPSS 26.0 to test the main effects of tuber region, developmental stage, and their interaction on starch content. The results are provided in Supplementary Tables S17 and S18.

To comprehensively assess the effects of cultivar, tuber region, developmental stage, and their interactions on starch accumulation, a three-way ANOVA was conducted on the combined dataset of both cultivars. Post hoc comparisons were performed using Tukey’s multiple-comparison test to identify significant differences among tuber regions within each developmental stage × cultivar combination (*p* < 0.05). In addition, pairwise comparisons between the two cultivars at each developmental stage were conducted via Welch’s independent samples *t*-test with Bonferroni correction for multiple comparisons.

### 2.4. Transcriptomic and Metabolomic Profiling

#### 2.4.1. Sample Preparation

Tuber samples at the S8 developmental stage from DX and D were subjected to combined metabolomic and transcriptomic sequencing, followed by qRT-PCR validation. Three independent biological replicates were used for all experiments, with each replicate consisting of a mixture of 10 individual tubers.

#### 2.4.2. RNA-Seq Library Construction and Transcriptomic Analysis

Total RNA was extracted from tuber samples using TRIzol reagent (Invitrogen, Waltham, CA, USA). The purity and integrity of RNA were assessed via 1% agarose gel electrophoresis, NanoDrop spectrophotometer (Thermo Scientific, Waltham, DE, USA), Qubit^®^ 2.0 Fluorometer (Life Technologies, Carlsbad, CA, USA) and Bioanalyzer 2100 system (Agilent Technologies, Santa Clara, CA, USA). Strand-specific RNA libraries were constructed with the NEBNext^®^ UltraTM Directional RNA Library Prep Kit for Illumina^®^ (NEB, Ipswich, MA, USA). Briefly, mRNA was fragmented using fragmentation buffer. First-strand cDNA was synthesized with random hexamer primers, followed by second-strand cDNA synthesis using DNA Polymerase I and dNTPs. Double-stranded cDNA was purified with AMPure XP beads, then subjected to end repair, A-tailing and adapter ligation. Qualified libraries were sequenced on the Illumina HiSeq platform at Maiwei Biotechnology Co., Ltd. (Wuhan, China).

Raw sequencing reads were trimmed and filtered using Trimmomatic v0.33 to remove adapters, low-quality bases and short reads, generating clean reads. Clean reads were mapped to the potato reference genome DM v6.1 (Solanum tuberosum DM1-3 516 R44 genome assembly version 6.1) using Hisat2 v2.1.0. Gene expression levels were quantified as TPM values with HTSeq v0.11.2. Differentially expressed genes (DEGs) were screened using DESeq2 v1.32.0, with the threshold of |log_2_Fold Change| ≥ 1 and *p*-value < 0.05.

#### 2.4.3. Untargeted Metabolomics Analysis

Untargeted metabolomic analysis was performed using liquid chromatography–tandem mass spectrometry (LC-MS/MS). Metabolomic samples were strictly paired with transcriptomic sequencing samples to ensure consistent sampling positions and tuber developmental stages, thus achieving temporal and spatial consistency between transcriptomic and metabolomic data. After thorough grinding, endogenous metabolites were extracted from fresh samples using a methanol–aqueous solution system. The supernatant was collected via low-temperature centrifugation, followed by filtration and purification to prepare test samples, which were subjected to LC-MS/MS detection under positive and negative ion modes separately. Raw mass spectrometry data were preprocessed sequentially including noise reduction, peak picking, peak alignment and retention time correction, followed by data normalization. Quality control (QC) pooled samples were inserted throughout the detection process to monitor instrument stability and assay repeatability. Metabolite identification was annotated against public databases including HMDB, Metlin and KEGG. Orthogonal partial least squares discriminant analysis (OPLS-DA) combined with univariate statistical analysis was adopted to screen differential metabolites (DMet) with the criteria: VIP ≥ 1, |log_2_FC| ≥ 1 and *p* < 0.05.

#### 2.4.4. Quantitative Real-Time PCR Validation

Quantitative real-time PCR (qRT-PCR) was performed for gene expression validation. First-strand cDNA was synthesized via one-step reverse transcription using PrimeScript RT Master Mix (Perfect Real Time, TaKaRa) following the manufacturer’s instructions. The reverse transcription program was set as 37 °C for 15 min, followed by enzyme inactivation at 85 °C for 5 s, and the obtained cDNA was stored at 4 °C for subsequent qRT-PCR detection. qRT-PCR amplification was carried out on a LightCycler 96 real-time PCR system (Roche) with TB Green Premix Ex Taq II (TaKaRa) using a two-step amplification protocol. The COX1 gene (GenBank ID: X83206.1) was used as the reference gene for internal normalization. The relative gene expression levels were calculated using the 2^−ΔΔCT^ method. Primer sequences for qRT-PCR are listed in Table S1.

#### 2.4.5. Tissue-Specific Expression Profiling of Candidate Genes

Tissue expression analysis of candidate genes was performed using the high-starch cultivar Atlantic. Uniform plants at the tuber bulking stage were sampled to collect six tissue types: flowers, functional leaves, stems, roots, tuber epidermis and tuber perimedullary region. Three independent biological replicates were collected for each tissue sample for subsequent gene expression quantification. Primer sequences for qRT-PCR are listed in Table S1.

## 3. Results

### 3.1. Quantification of Starch Content

**Figure 1:**
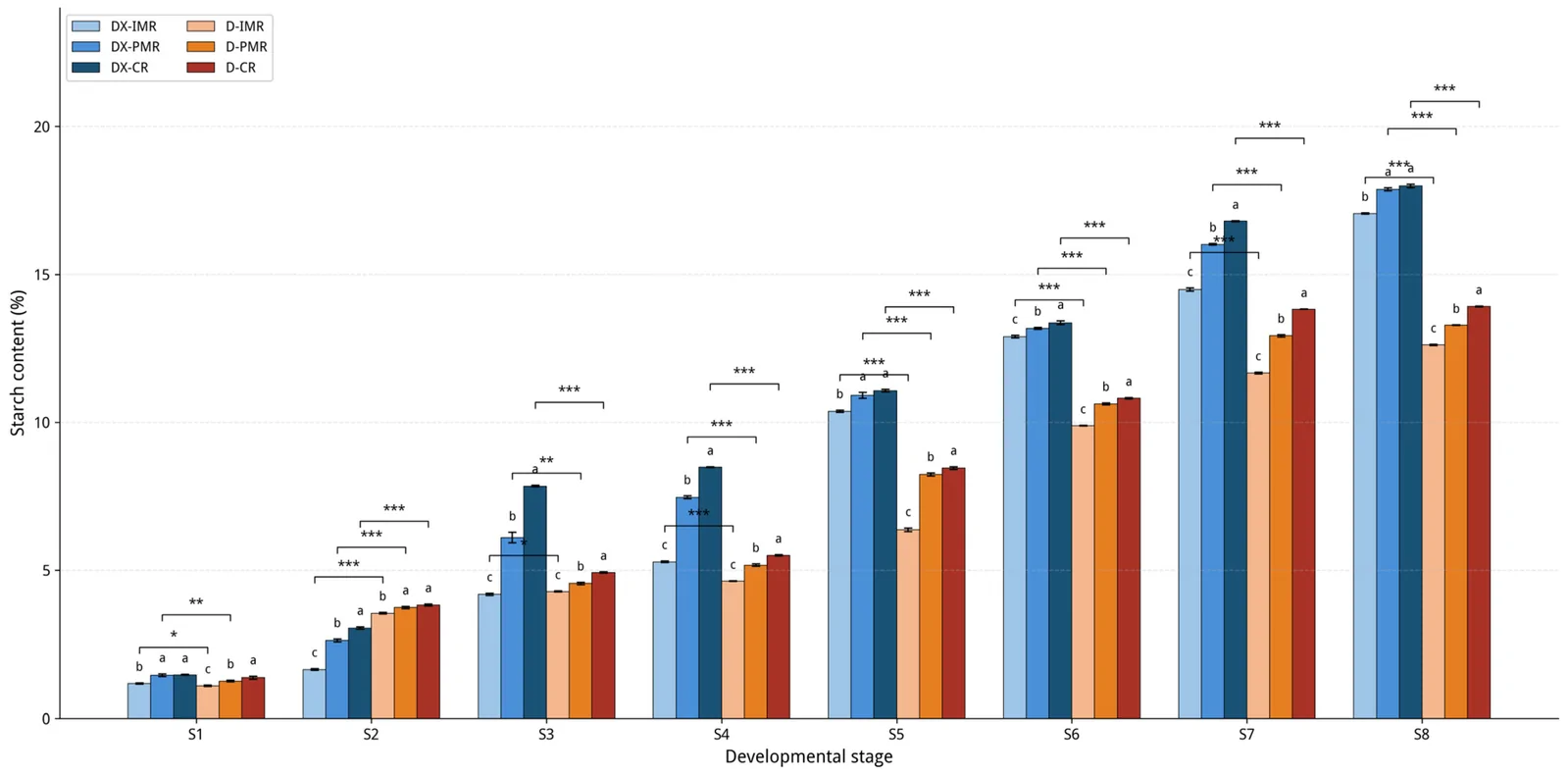
**Dynamic changes in starch content across three tuber tissues of the high-starch cultivar DX and the low-starch cultivar D during eight developmental stages (S1–S8).** DX-IMR, DX-PMR, and DX-CR represent the inner medullary region, perimedullary region, and cortex of Atlantic tubers, respectively; D-IMR, D-PMR, and D-CR correspond to the three tuber regions of Dingshu No. 1. Error bars denote the standard deviation (SD) of three biological replicates. A three-way ANOVA was performed to test the effects of cultivar, tuber region, developmental stage, and their interactions (Tables S17 and S18). Tukey’s multiple-comparison test was used for post hoc comparisons; significant differences (*p* < 0.05) among tissues within each developmental stage × cultivar combination are indicated by lowercase letter markers (a, b, c) on bars. Inter-cultivar significant differences at each developmental stage were determined using Welch’s independent samples *t*-test with Bonferroni correction. * *p* < 0.05, ** *p* < 0.01, *** *p* < 0.001; ns, no significant difference.

Total starch contents in three tuber regions—inner medullary region (IMR), perimedullary region (PMR), and cortex (CR)—of the high-starch cultivar DX and the low-starch cultivar D were quantified across eight sequential developmental stages (S1–S8). Detailed measurements are provided in Tables S2 and S3, and the combined dynamic profiles of both cultivars are presented in Figure 1. Individual cultivar-specific starch accumulation curves and the corresponding two-way ANOVA results are available in Supplementary Tables S17 and S18.

Both cultivars exhibited a conserved biphasic temporal pattern of starch deposition, comprising a slow accumulation phase (S1-S4) followed by a rapid accumulation phase (S5–S8) (Figure 1). In DX, starch contents during the slow phase increased from 1.17%, 1.45%, and 1.46% to 5.28%, 7.46%, and 8.48% in IMR, PMR, and CR, respectively, representing absolute increments of 4.11, 6.01, and 7.02 percentage points. During the subsequent rapid phase, starch accumulation accelerated markedly, reaching 17.05% (IMR), 17.87% (PMR), and 18.00% (CR) at S8, with increments of 11.77, 10.41, and 9.52 percentage points relative to the onset of the rapid phase. The low-starch cultivar D followed an analogous biphasic trajectory, but with substantially lower starch concentrations throughout development. During the slow phase, starch levels in D rose from 1.10%, 1.25%, and 1.37% to 4.63%, 5.17%, and 5.50% in IMR, PMR, and CR, respectively. In the rapid phase, starch contents increased by 6.06, 8.11, and 8.39 percentage points, attaining final values of 12.61%, 13.27%, and 13.89% at S8.

Spatially, both cultivars consistently displayed a starch gradient of CR > PMR > IMR at every developmental stage (Figure 1). In DX, the concentration gap between CR and IMR widened progressively from 0.29 percentage points at S1 to 0.62 at S5 and 0.95 at S8, indicating amplifying regional disparities during tuber maturation. This cortical predominance aligns with previous reports and may reflect earlier cell differentiation and more advanced amyloplast development in cortical tissues. Cultivar D exhibited the same CR > PMR > IMR gradient but with narrower inter-regional differences, consistent with its overall lower starch biosynthetic capacity.

Direct inter-cultivar comparison revealed genotype-specific accumulation dynamics (Figure 1). At the early developmental stage S2, starch content and accumulation rates in all three tuber regions of D were significantly higher than those in DX (*p* < 0.001), indicating that D initiates starch synthesis more rapidly during early tuber expansion. However, from S4 onward, DX surpassed D in starch accumulation rate across all tissues, and the inter-cultivar starch content gap widened continuously with tuber growth. At the mature harvest stage S8, starch contents in DX exceeded those in D by 4.44, 4.60, and 4.11 percentage points in IMR, PMR, and CR, respectively (all *p* < 0.001), demonstrating DX’s superior capacity for starch synthesis and storage.

A three-way ANOVA was performed to assess the effects of cultivar (V), tuber region (R), developmental stage (S), and their interactions on starch content (Table S1). The main effects of tuber region (F = 25.64, *p* < 0.001) and developmental stage (F = 30,664.40, *p* < 0.001) were highly significant, while the cultivar effect was significant (F = 6.52, *p* = 0.012). Significant two-way interactions were observed for cultivar × stage (F = 1876.04, *p* < 0.001) and region × stage (F = 225.26, *p* < 0.001), whereas the cultivar × region interaction was not significant (F = 2.99, *p* = 0.055). The three-way interaction cultivar × region × stage was highly significant (F = 418.45, *p* < 0.001), indicating that the spatiotemporal pattern of starch accumulation differed between the two cultivars. Post hoc Tukey’s multiple-comparison tests confirmed that, within each developmental stage × cultivar combination, CR consistently exhibited significantly higher starch content than PMR and IMR, while PMR was generally significantly higher than IMR (*p* < 0.05); these differences are denoted by lowercase letter markers on the bars in Figure 1.

Collectively, high- and low-starch tetraploid potato cultivars shared a conserved biphasic temporal accumulation rhythm and a CR > PMR > IMR spatial gradient. However, cultivar genotype profoundly influenced starch accumulation efficiency, final starch concentration, and the magnitude of intra-tuber spatial differentiation, ultimately giving rise to the distinct high-starch (DX) and low-starch (D) phenotypes.

### 3.2. Analysis of Transcriptomic and Metabolomic Sequencing Results

Consistent physiological data revealed universal CR > PMR > IMR starch distribution across cultivars, with maximal concentration divergence between CR and IMR. To precisely resolve the molecular mechanisms underlying tissue-specific starch accumulation within individual cultivars, CR and IMR exhibiting the most prominent starch content gaps were selected for subsequent multi-omics analysis.

#### 3.2.1. Transcriptomic Analysis

A total of 655,900,816 raw reads were generated via Illumina NovaSeq sequencing. After adapter trimming and low-quality read filtering, 639,605,340 high-quality clean reads (95.94 Gb) were retained. The average Q20 value (base call accuracy ≥ 99%) reached 98.86%, with an average GC content of 42.78%, demonstrating outstanding sequencing quality. Approximately 77.75% of clean reads from all samples were uniquely mapped to the potato reference genome, ensuring high read utilization and providing robust data resources for downstream transcript quantification, DEG screening and functional annotation (detailed data are provided in Tables S4–S11).

**Figure 2:**
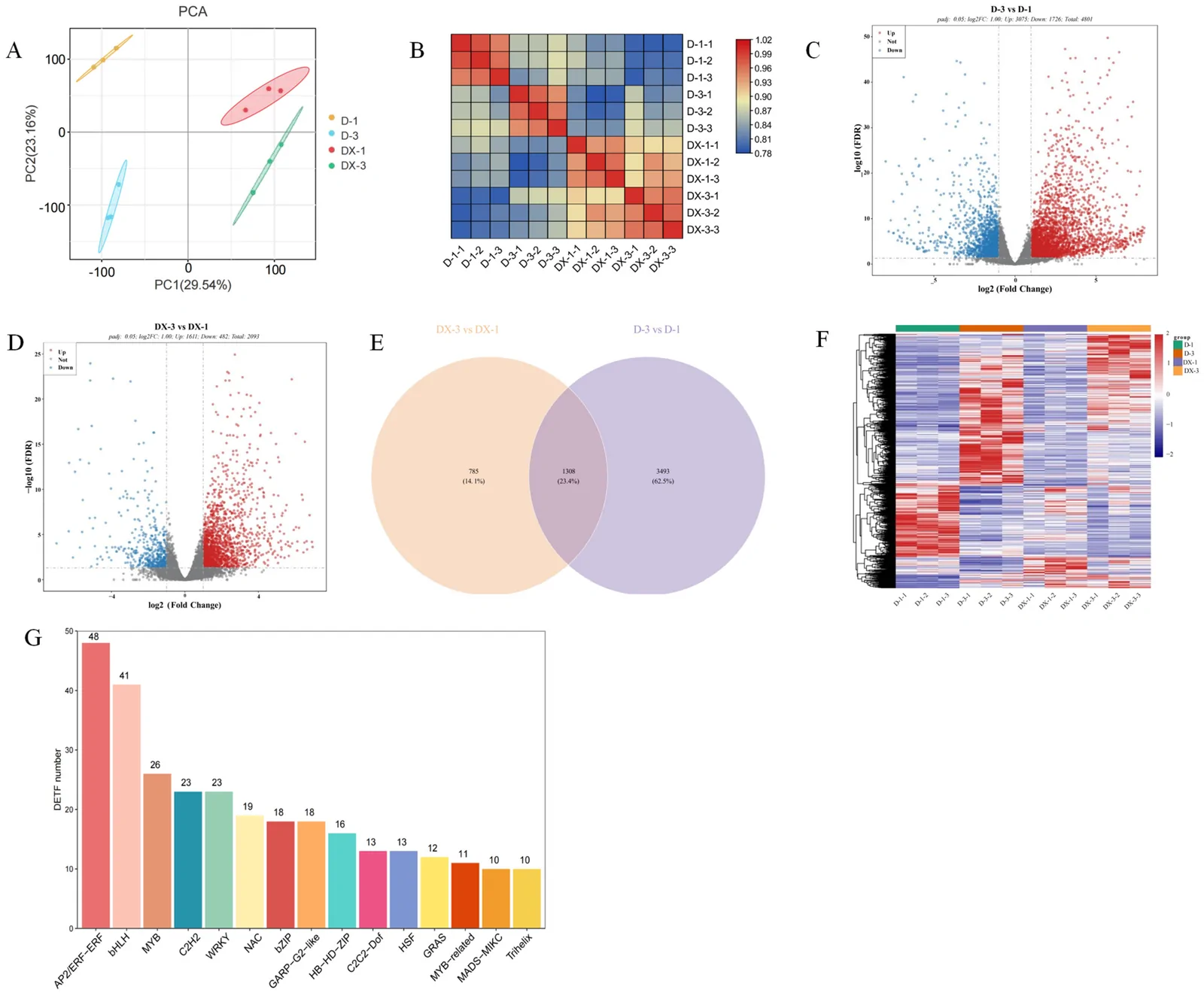
**Transcriptomic analysis of starch accumulation in potato tubers.** (**A**) PCA of mRNA transcript profiles; (**B**) Pearson correlation heatmap of transcriptome samples; (**C**,**D**) volcano plots of DEGs for D-3 vs. D-1 and DX-3 vs. DX-1; (**E**) Venn diagram of DEGs from two comparative groups; (**F**) hierarchical clustering heatmap of all non-redundant DEGs; (**G**) bar chart illustrating the top 15 TF families with differential expression.

Principal component analysis (PCA) was performed to characterize global transcriptional variation across samples. Clear spatial separation was observed among four sample groups (D-1, D-3, DX-1, DX-3) in the PCA plot, with tight clustering of three biological replicates within each group (Figure 2A). These results revealed profound transcriptional reprogramming differences between the two cultivars, as well as between the cortex and inner medulla within each cultivar, and verified excellent sample reproducibility.

Pearson correlation heatmaps further confirmed extremely high correlation coefficients among biological replicates within each group and relatively low correlations between distinct sample clusters (Figure 2B), independently validating the reliability and consistency of the sequencing data to support subsequent DEG identification.

DEGs were screened using DESeq2 with thresholds of adjusted *p* < 0.05 and |log_2_FC| > 1 for two pairwise comparisons: D-3 vs. D-1 and DX-3 vs. DX-1, visualized via volcano plots (Figure 2C,D).

For D-3 vs. D-1, 4801 DEGs were identified, comprising 3075 upregulated and 1726 downregulated transcripts. The predominance of upregulated genes indicated widespread transcriptional activation from the inner medulla to the cortex in D. For DX-3 vs. DX-1, 2093 DEGs were detected, including 1611 upregulated and 482 downregulated genes. Although upregulated transcripts remained dominant, the total number of DEGs was substantially lower than that in D, and the count of downregulated genes was merely 27.9% of that observed in D, demonstrating milder transcriptional divergence between cortical and medullary tissues in Atlantic, and divergent intrinsic transcriptional regulatory programs across cultivars.

Venn analysis of DEGs from the two comparative groups identified 1308 shared DEGs alongside cultivar-specific differentially expressed transcripts, yielding a total of 5586 non-redundant DEGs (Figure 2E). This result indicated both conserved transcriptional responses and cultivar-unique regulatory machinery governing tissue differentiation in potato tubers. Hierarchical clustering heatmaps of all non-redundant DEGs revealed highly uniform expression patterns within each sample group and striking transcriptional divergence between groups, especially between the two cultivars (Figure 2F), further substantiating systematic transcriptional differentiation across cultivars and tuber anatomical zones.

Transcription factors (TFs) act as central regulators of gene expression; thus, TF annotation and enrichment analysis were performed on the 5586 non-redundant DEGs, identifying 432 differentially expressed TFs in total (Figure 2G). Multiple TF families exhibited significant enrichment: the AP2/ERF family represented the largest cohort with 48 members, followed by bHLH (41), MYB (26), C2H2 (23) and WRKY (23). Functional profiles of core TF families are summarized in Figure 2G. Additional markedly enriched families included NAC (19), bZIP (18), GARP-G2-like (18), HB-HD-ZIP (16), C2C2-Dof (13), HSF (13) and GRAS (13). These enriched TF families likely function as master modulators of tissue-specific gene expression within tubers, and their differential abundance constitutes a critical molecular basis for transcriptional divergence between cortex and inner medulla in the two cultivars, offering core TF targets for dissecting regulatory networks of regional starch accumulation.

Gene Ontology (GO) and Kyoto Encyclopedia of Genes and Genomes (KEGG) pathway enrichment analyses were conducted on DEGs from D-3 vs. D-1 and DX-3 vs. DX-1 to systematically resolve functional differentiation and metabolic regulatory disparities between cortical and medullary tissues in the two cultivars.

For D-3 vs. D-1, GO enrichment revealed moderate enrichment of DEGs in carbohydrate biosynthetic biological processes, notably nucleotide sugar and carbohydrate biosynthesis. At the molecular function level, gene sets associated with enzyme inhibitor and peptidase inhibitor activity exhibited far stronger enrichment than sugar transmembrane transporter activity.

KEGG enrichment corroborated these observations: DEGs enriched in secondary metabolite biosynthesis, amino acid metabolism and plant hormone signal transduction displayed far higher significance and abundance than those involved in starch and sucrose metabolism. Although core carbon metabolic pathways (nucleotide sugar, amino sugar, starch/sucrose metabolism) were moderately enriched in D-3, their enrichment intensity remained limited. Lipid metabolism and ABC transporter-mediated substrate translocation pathways were also moderately activated in cortical tissues of D. Collectively, these data suggested that while carbon metabolism and sugar transport are moderately enhanced in D’s cortex relative to its medulla, most differentially expressed transcripts prioritize secondary defense and stress-resistance pathways, leading to substantial carbon flux diversion away from starch biosynthesis. This carbon partitioning bias represents a key molecular mechanism underlying the weak starch accumulation capacity of the low-starch cultivar D.

**Figure 3:**
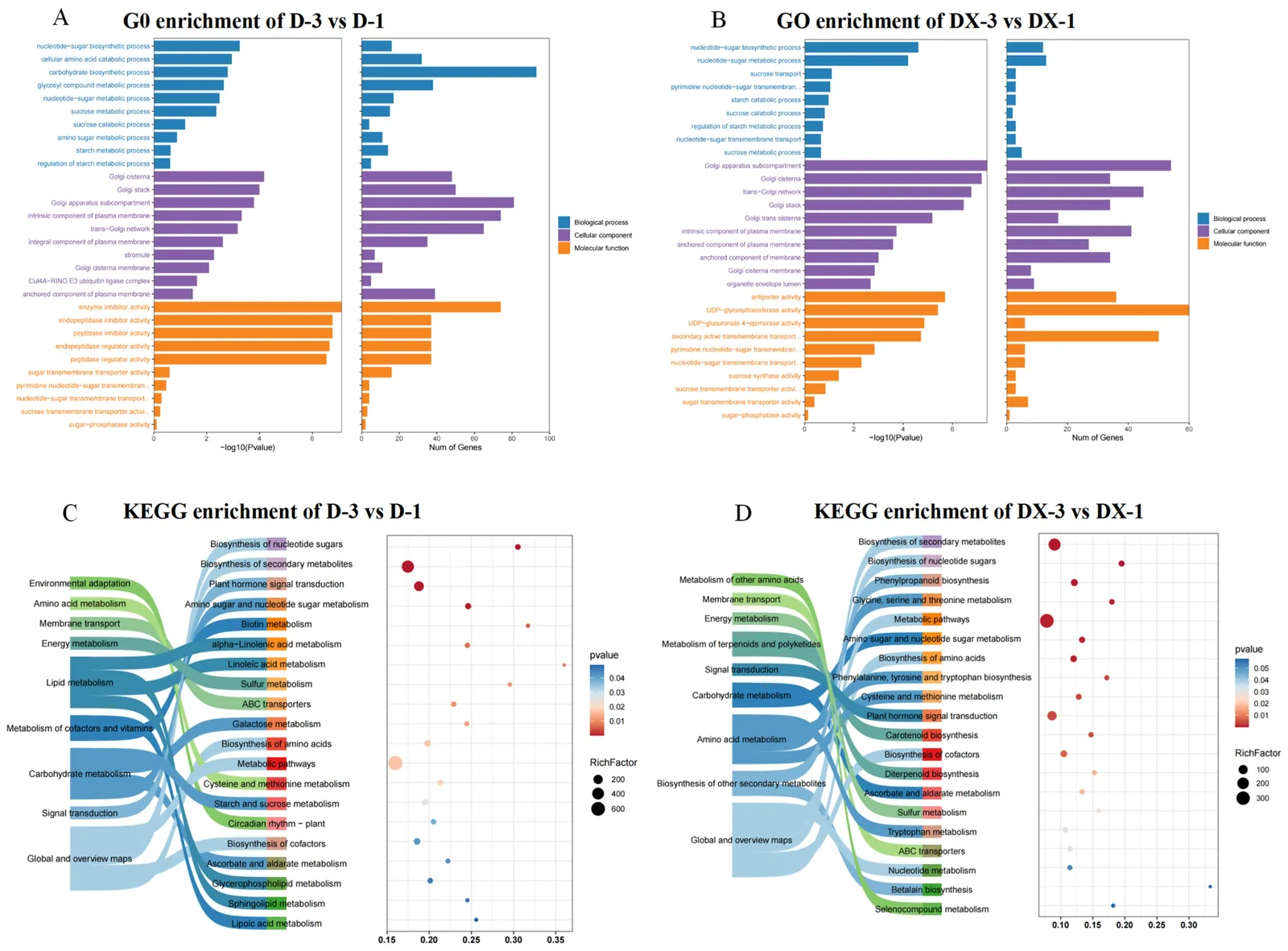
**Functional enrichment analyses reveal distinct biological processes and metabolic pathways associated with differential gene expression.** (**A**,**C**) GO enrichment analyses of DEGs for D-3 vs. D-1 and DX-3 vs. DX-1; (**B**,**D**) KEGG pathway enrichment analyses of DEGs for D-3 vs. D-1 and DX-3 vs. DX-1. In the KEGG panels, horizontal bars represent −log_10_-transformed *p*-values, with bar colors indicating KEGG pathway categories. The number of differentially expressed genes enriched in each pathway is annotated as “n=“ on the bars. The red dashed line indicates the significance threshold of *p* = 0.05.

GO and KEGG enrichment analyses for DX-3 vs. DX-1 uncovered highly conserved regulatory patterns relative to D. DEGs in Atlantic’s cortex were significantly enriched in core carbohydrate biosynthetic pathways (starch/sucrose metabolism, nucleotide sugar metabolism) and sugar/nucleotide sugar transmembrane transport functions (Figure 3). Phenylpropanoid secondary metabolism, fundamental energy and amino acid metabolism, and ABC transporter pathways were also robustly activated in DX-3, demonstrating that Atlantic’s cortex drives starch synthesis and deposition via coordinated activation of carbon biosynthesis, substrate transport, primary metabolism and secondary metabolism, ultimately generating the phenotype of higher cortical starch concentration relative to the inner medulla.

In summary, the cortex tissues of both cultivars establish a molecular framework for efficient starch biosynthesis and accumulation via coordinated activation of core carbohydrate pathways, enhanced substrate transport, and synergistic primary and secondary metabolic reprogramming, representing a highly conserved regulatory strategy across high- and low-starch potato germplasm. Tissue-specific metabolic differentiation within tubers constitutes an intrinsic determinant of spatial starch disparities, and the enrichment results provide essential functional evidence for further dissection of tissue-unique starch regulatory pathways and hub-gene mining.

#### 3.2.2. Metabolomics Profiling

Untargeted metabolomics was performed across distinct tuber zones of high- and low-starch cultivars to characterize tissue-specific metabolite signatures (detailed data are provided in Table S12).

**Figure 4:**
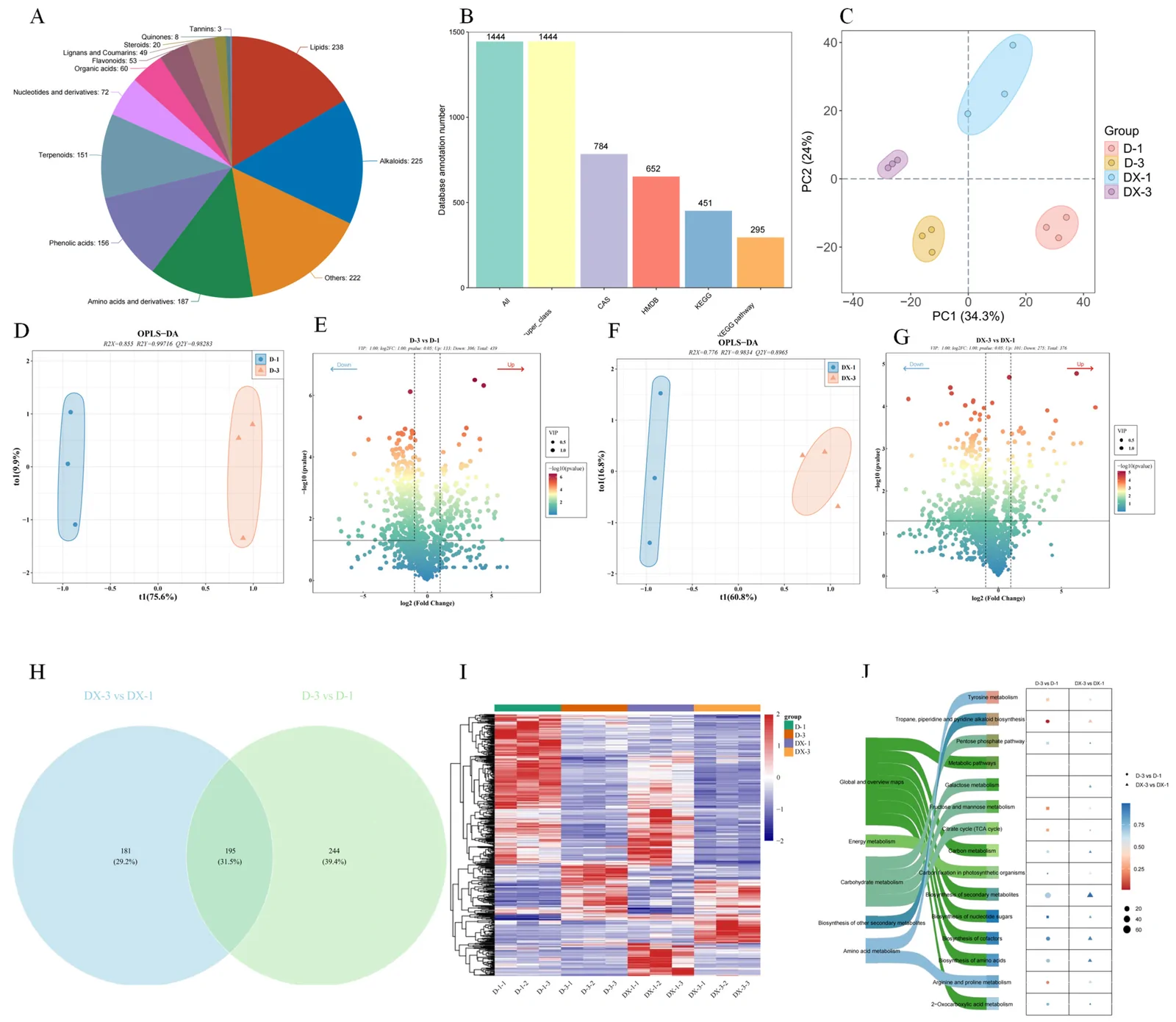
**Global metabolomic profiling reveals distinct metabolic signatures and differential metabolite accumulation across conditions.** (**A**) Pie chart of metabolite classification; (**B**) bar plot of metabolite database annotation statistics; (**C**) PCA of metabolomic profiles across four sample groups; (**D**,**F**) OPLS-DA score plots for pairwise comparisons; (**E**,**G**) volcano plots of DMet for two comparative groups; (**H**) Venn diagram of DMet from D-3 vs. D-1 and DX-3 vs. DX-1; (**I**) clustering heatmap of all identified DMet; (**J**) bubble plot of KEGG pathway enrichment for DMet.

In total, 1444 metabolites were annotated across DX-3, DX-1, D-3 and D-1 samples, distributed into 13 major biochemical categories. Lipids (238), alkaloids (225) and other unclassified metabolites (222) constituted the three dominant classes, alongside amino acids and derivatives (187), phenolic acids (156) and terpenoids (151) (Figure 4A,B). Comprehensive metabolite annotation against multiple public databases was completed to facilitate downstream functional interpretation.

PCA demonstrated tight clustering of biological replicates within each group and clear inter-group separation, indicating profound inherent differences in metabolomic profiles across tuber tissues and cultivars. This metabolomic trend was highly consistent with transcriptomic observations, mutually validating the experimental reliability (Figure 4C).

OPLS-DA models were constructed for DX-3 vs. DX-1 and D-3 vs. D-1 to accurately quantify inter-group metabolic divergence (Figure 4D,F). Model performance metrics indicated robust fitting and predictive power: for D-3 vs. D-1, R^2^X = 0.855, R^2^Y = 0.997, Q^2^Y = 0.982; for DX-3 vs. DX-1, R^2^X = 0.778, R^2^Y = 0.983, Q^2^Y = 0.896. Near-unity R^2^ values and high Q^2^Y scores confirmed the suitability of the models for reliable DEM screening.

DMet were filtered under thresholds |log_2_FC| > 0.584 and VIP > 1. For D-3 vs. D-1, 439 DMet were detected, including 133 upregulated and 306 downregulated metabolites, with downregulated compounds dominating differential metabolic shifts. In contrast, DX-3 vs. DX-1 yielded 376 DMet, comprising 275 upregulated and 106 downregulated metabolites, with upregulated metabolites exceeding downregulated counterparts by more than twofold—revealing fundamentally distinct tissue-specific metabolic regulatory modes between the two cultivars (Figure 4E,G).

Venn analysis identified 195 overlapping DMet shared by both comparative groups, alongside cultivar-specific differential metabolites, reflecting both universal and unique metabolic remodeling responses governing cortical–medullary differentiation (Figure 4H). Hierarchical clustering heatmaps of all DMet intuitively visualized divergent metabolite-accumulation patterns across experimental groups (Figure 4I).

KEGG enrichment of DMet highlighted prominent enrichment in core pathways, including general metabolic networks, galactose metabolism and the pentose phosphate pathway, alongside carbon fixation in photosynthetic organisms, secondary metabolite biosynthesis, the tricarboxylic acid (TCA) cycle, fructose and mannose metabolism, and amino acid metabolism (tyrosine, arginine and proline). These results demonstrate that metabolic disparities between high- and low-starch potato tubers span primary biosynthesis, energy homeostasis, secondary metabolism and amino acid turnover (Figure 4J).

Separate pathway enrichment analyses for the two cultivar comparisons pinpointed starch and sucrose metabolism, the TCA cycle, fructose/mannose metabolism, and amino sugar and nucleotide sugar metabolism as the primary divergent carbon metabolic pathways, with 12 core sugar-related DMet selected for systematic inter-group comparison.

**Figure 5:**
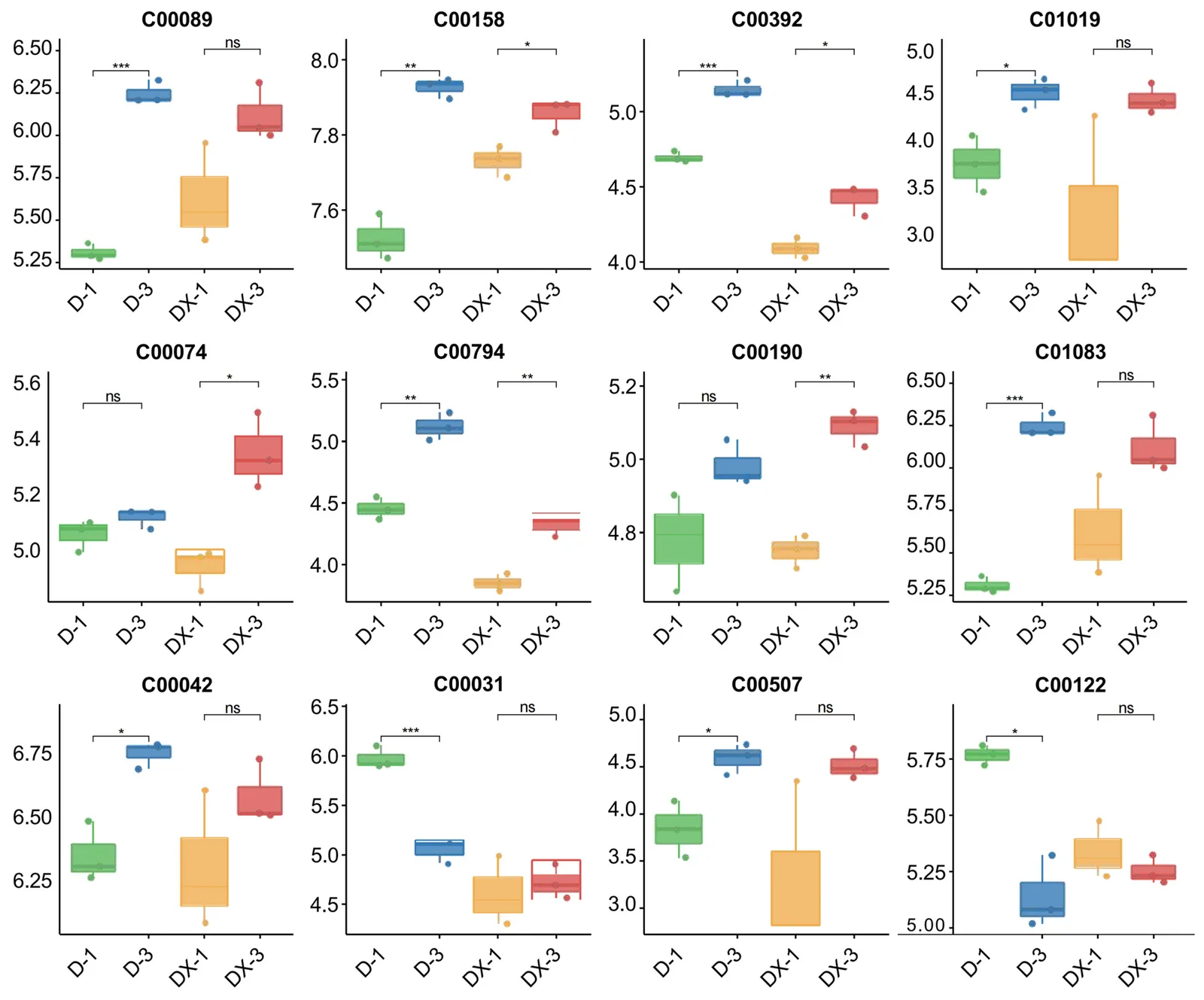
**Accumulation profiles of 12 core sugar-related DMet across four sample groups**. Box-and-whisker plots illustrate the relative expression levels of 12 representative genes across four tuber regions (D-1, D-3, DX-1, DX-3). Colours correspond to different tissue sections. Statistical significance is indicated: * *p* < 0.05, ** *p* < 0.01, *** *p* < 0.001; ns, no significant difference.

For D-3 vs. D-1, the 12 sugar-associated DMet exhibited extreme tissue-specific accumulation disparities: 10 metabolites (C00089, C00158, C00392, C01019, C00190, C01083, C00042, C00507, C00794, C00074) accumulated at significantly higher concentrations in the cortex, while only two metabolites (C00031, C00122) were enriched in the inner medulla. These data indicated drastically polarized carbon metabolic activity in D, with hyperactive sugar metabolism confined exclusively to cortical tissues (Figure 5).

For DX-3 vs. DX-1, tissue-specific disparities of the 12 sugar DMet were largely attenuated. Only one metabolite (C00074) displayed significant cortical enrichment relative to the inner medulla, whereas the remaining 11 metabolites showed non-significant concentration differences between DX-1 and DX-3. Most sugar metabolites maintained globally elevated basal abundance in Atlantic, with balanced accumulation across cortical and medullary tissues, characteristic of uniform and coordinated carbon metabolism throughout tubers of the high-starch cultivar.

Comparative integration of DX and D metabolomic data revealed distinct carbon partitioning strategies: the low-starch cultivar D relies solely on cortical metabolic activation to drive sugar flux, but insufficient medullary metabolic capacity restricts substrate supply for starch synthesis. In contrast, the high-starch cultivar Atlantic sustains globally activated and homogenized carbon metabolism across all tuber zones, providing stable and abundant precursors for sustained starch biosynthesis.

Collectively, sugar metabolic pathways exhibit extreme tissue polarization in low-starch potato tubers, while high-starch cultivars maintain uniformly activated, balanced carbon metabolism across anatomical regions. This divergent metabolite accumulation pattern constitutes a primary driver of cultivar- and tissue-specific starch concentration disparities.

#### 3.2.3. Integrated Transcriptome–Metabolome Analysis

**Figure 6:**
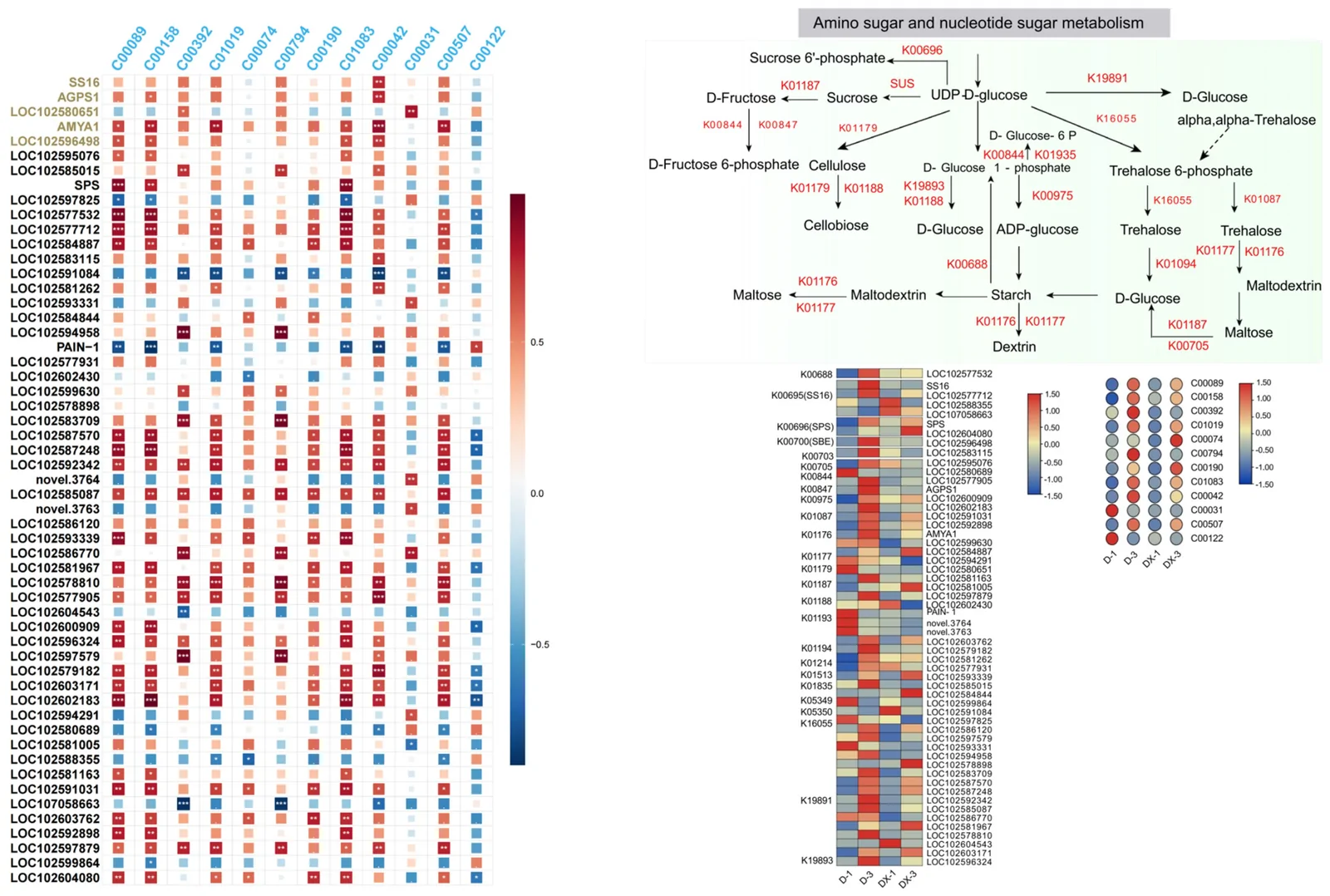
Transcript profiles of amino sugar and nucleotide sugar metabolism-related genes in potato tuber tissues. Heatmaps show relative expression of sugar-metabolism and KO-annotated genes across four tuber spatial regions (D-1, D-3, DX-1, DX-3). Red denotes high gene expression and blue denotes low expression. The schematic illustrates amino sugar and nucleotide sugar metabolic pathways. * *p* < 0.05, ** *p* < 0.01, *** *p* < 0.001, indicating statistically significant differences.

Integrated multi-omics analysis was conducted to resolve intrinsic correlations between starch biosynthetic gene expression and metabolite accumulation, and to define molecular signatures governing tissue-specific starch deposition within potato tubers (detailed data are provided in Tables S13 and S14). Within the KEGG starch and sucrose metabolic pathway (ko00500), 25 differential structural genes encoding key starch biosynthetic enzymes (sucrose synthases, starch synthases, α-amylases, phosphoglucomutases, etc.) were identified, corresponding to 55 potato homologous transcripts (filtered to remove low-expression genes with average FPKM < 1). Hub differential genes within this pathway included SS16, AGPS1, SPS, AMYA1, LOC102580651 and LOC102596498 (Figure 6). Correlation analysis linking these starch-related DEGs and DMet clearly delineated divergent carbon flux partitioning mechanisms underlying differential starch accumulation between high-starch DX (DX-1/DX-3) and low-starch D (D-1/D-3) tubers.

Pathway mapping identified UDP-D-glucose as a central carbon hub, which can be channeled toward starch biosynthesis (via ADP-glucose and starch synthases) or diverted into competing branches for cellulose, trehalose and sucrose production. Combined gene and metabolite heatmaps revealed cultivar-specific regulatory divergence: in low-starch D, key starch biosynthetic enzymes (K00975/AGPase, K01176/amylase) were transcriptionally repressed in the cortex (D-3), while genes driving competing carbon branches (K01179/cellulose synthase, K16055/trehalose synthase) were significantly upregulated. Simultaneously, carbon-competing sugar metabolites (C00158, C00392) massively accumulated in D’s cortex, whereas the inner medulla (D-1) lacked sufficient carbon metabolic capacity, generating a polarized “cortex carbon diversion, medulla substrate deficiency” phenotype. In contrast, Atlantic exhibited balanced expression of starch biosynthetic pathway genes across the cortex (DX-3) and the inner medulla (DX-1), accompanied by stable supply of starch precursor metabolites (ADP-glucose). Genes mediating competing carbon branches were maintained at low expression levels, establishing a whole-tuber coordinated carbon supply system prioritizing starch biosynthesis. These integrated multi-omics results were fully consistent with independent transcriptomic and metabolomic enrichment analyses, confirming that carbon flux diversion toward amino sugar and nucleotide sugar metabolism represents a major constraint on starch accumulation in low-starch cultivars, whereas high-starch germplasm achieves efficient uniform starch deposition via suppression of competing carbon branches and stable provision of starch precursors.

From the 25 differential structural genes identified in ko00500, 18 representative transcripts (SS16, AGPS1, SPS, AMYA1, PAIN-1 and 13 LOC-family homologous genes) were selected for detailed expression characterization to resolve precise synergistic regulatory relationships between DEGs and DMet within the pathway, illustrating directional transcriptional control over metabolite synthesis and storage compound accumulation.

SS16 and SPS catalyze reciprocal sucrose synthesis and degradation to generate UDP-D-glucose substrates for starch production. AGPS1 encodes the rate-limiting enzyme of starch biosynthesis, driving ADP-glucose formation and amylose/amylopectin chain elongation. LOC102593331 (TPS) encodes trehalose-6-phosphate synthase, governing carbon flux allocation and sugar signal transduction; it coordinates with linkage genes LOC102596498, LOC102577712 and LOC102577532 to modulate sugar precursor partitioning. AMYA1 and PAIN-1 negatively regulate starch accumulation by mediating starch hydrolytic breakdown. Hierarchical regulatory relationships between LOC102577931 and core functional genes (SS16, AGPS1), as well as targeted correlations between the 18 hub transcripts and sugar metabolites, provide foundational evidence for constructing integrated molecular regulatory networks governing potato starch biosynthesis.

Combined tissue-specific gene expression and gene-metabolite correlation analyses revealed divergent regulatory cascades between cultivars. In high-starch Atlantic (DX-1, DX-3), positive starch biosynthetic genes (SS16, AGPS1, SPS) and sugar transporter genes were significantly upregulated, while starch hydrolysis genes (AMYA1, PAIN-1) and carbon diversion gene LOC102593331 (TPS) were transcriptionally suppressed. This cascade established a coordinated regulatory model featuring efficient upstream substrate accumulation, robust midstream enzymatic catalysis, sustained downstream starch polymerization, inhibited starch degradation and minimized carbon flux diversion, directing the majority of fixed carbon toward starch biosynthesis.

Conversely, this coordinated regulatory module was severely disrupted in low-starch D (D-1, D-3): positive starch synthetic genes were weakly expressed, hydrolytic genes AMYA1 and PAIN-1 maintained constitutively high expression, and carbon diversion gene LOC102593331 (TPS) was significantly upregulated. Collectively, these transcriptional shifts led to insufficient starch precursor supply, aggravated starch catabolism and massive carbon redirection into secondary metabolic branches, ultimately suppressing total tuber starch accumulation. Tissue- and cultivar-specific differential expression of these 18 hub structural genes confirmed that spatiotemporal transcriptional regulation constitutes the core molecular driver of starch concentration divergence across potato tuber anatomical zones.

#### 3.2.4. Tissue-Specific Expression Results of Candidate Genes

**Figure 7:**
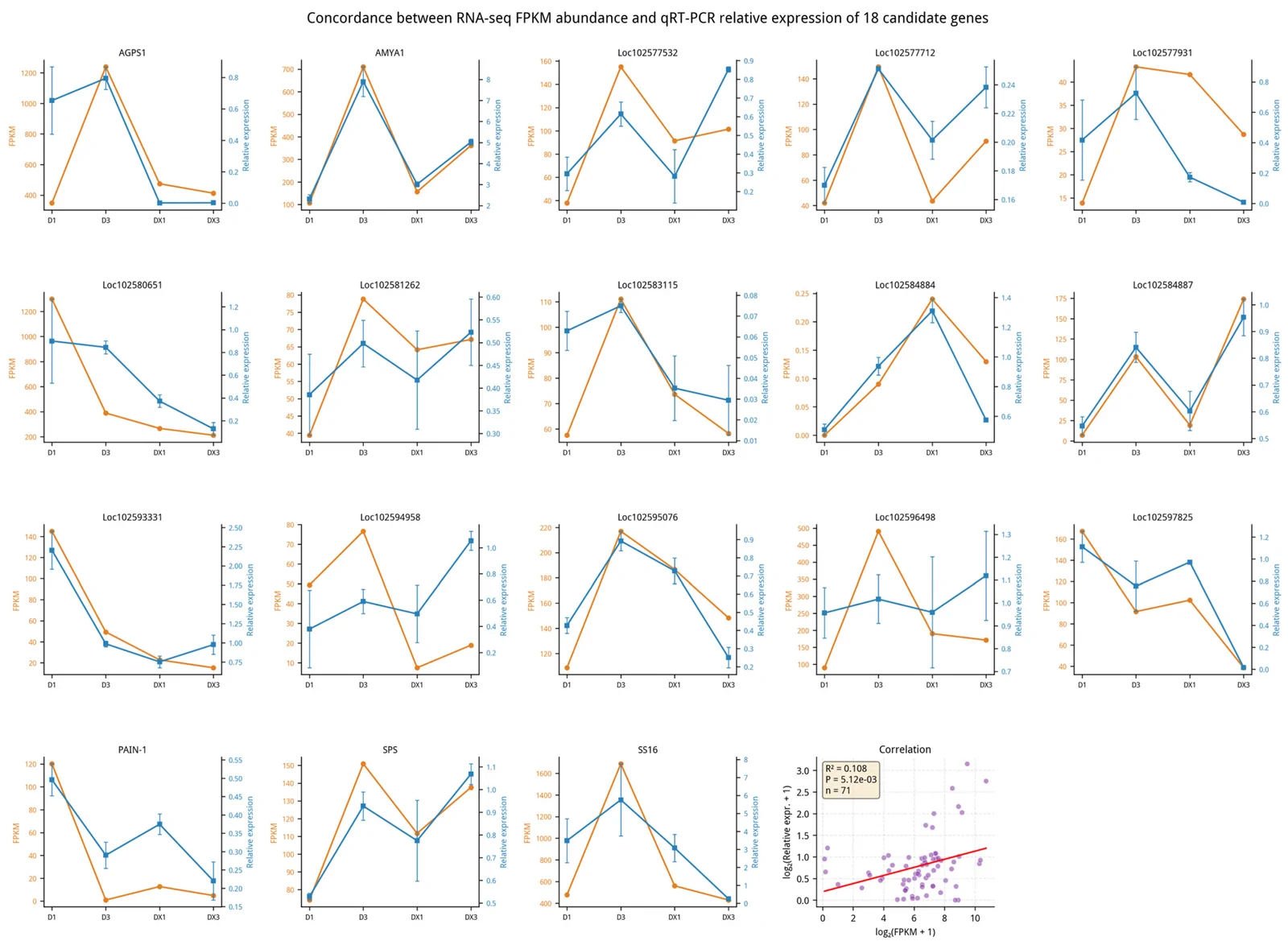
**Concordance between RNA-seq FPKM abundance and qRT-PCR relative expression of 18 candidate genes.** Each panel shows the expression profile of one gene across four conditions: D1 and D3 (low-starch cultivar Dingshu No. 1 at developmental stages 1 and 3), and DX1 and DX3 (high-starch cultivar Atlantic at developmental stages 1 and 3). Orange lines with circle markers represent RNA-seq FPKM values (left y-axis); blue lines with circle markers represent qRT-PCR relative expression determined by the 2^−ΔΔCt^ method (right y-axis). Error bars on qRT-PCR data indicate standard deviation (SD) of three biological replicates. The bottom-right panel shows the Pearson correlation between log_2_-transformed RNA-seq FPKM and qRT-PCR relative expression across all genes and conditions (n = 71), withR^2^ = 0.108 and *p* = 5.12 × 10^−3^, indicating a significant positive correlation between the two platforms.

qRT-PCR was performed on the 18 hub DEGs associated with starch and sucrose metabolism (ko00500) to validate transcriptomic sequencing accuracy and reinforce multi-omics correlation results (detailed data are provided in Table S15). Relative expression trends quantified via qRT-PCR were generally consistent with RNA-seq FPKM values (Figure 7), fully verifying the authenticity and reliability of our transcriptomic dataset.

Starch accumulation in potato tubers represents a complex biological process coordinated by multiple genes, pathways and tissues. Based on integrated regional multi-omics analyses, three evolutionarily conserved candidate genes were prioritized for functional characterization: LOC102593331 (TPS), LOC102584887 (AMY) and LOC102580651 (EN). RT-qPCR was utilized to quantify tissue-specific transcript abundance of EN, AMY and TPS across six Atlantic tissues: flowers, functional leaves, stems, roots, tuber epidermis and tuber perimedullary region (detailed data are provided in Table S16).

**Figure 8:**
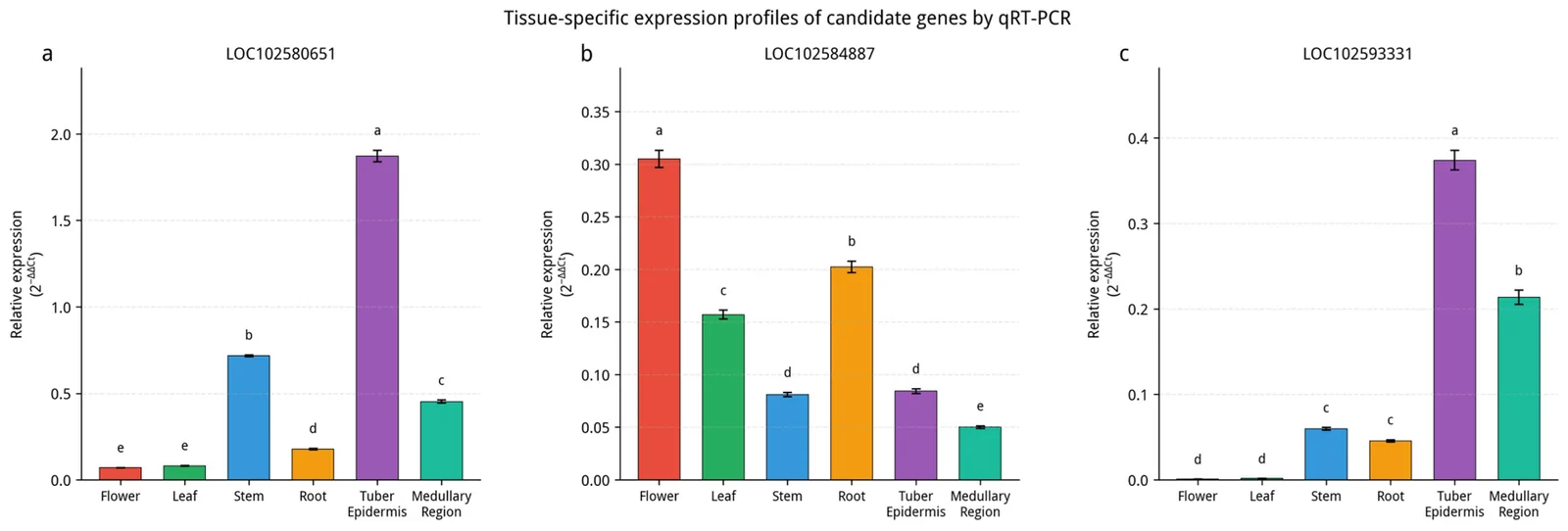
Tissue-specific expression profiles of three candidate genes determined by quantitative real-time PCR (qRT-PCR). (**a**) LOC102580651, (**b**) LOC102584887, and (**c**) LOC102593331 transcript levels in six tissues—flower, leaf, stem, root, tuber epidermis, and medullary region. Relative expression was calculated using the 2^−ΔΔCt^ method. Data are presented as mean ± standard deviation (SD) of three biological replicates. One-way ANOVA followed by Tukey’s multiple-comparison test was performed for each gene; significant differences (*p* < 0.05) among tissues are indicated by lowercase letters (a–e) above the bars.

Relative expression levels of the three candidate genes across six Atlantic tissues are summarized in Figure 8, revealing pronounced tissue-specific expression patterns with distinct transcriptional partitioning. LOC102580651 (EN) exhibited maximal transcript abundance in tuber epidermis. LOC102584887 (AMY) was predominantly expressed in aerial vegetative organs (flowers, leaves, roots) and was sharply downregulated in tuber storage tissues. LOC102593331 (TPS) displayed tuber-preferential expression, with negligible transcript accumulation in photosynthetic aerial organs.

## 4. Discussion

Starch accumulation exhibits obvious spatial heterogeneity in different anatomical regions of potato tubers, with consistently higher starch contents in cortical tissues than in inner medulla tissues. This tissue-specific starch gradient is not determined solely by cultivar genetic background, but is potentially shaped by tissue-specific transcriptional reprogramming and metabolic remodeling. In the present study, we performed integrated transcriptomic and metabolomic analyses of tuber cortex and inner medulla tissues from the high-starch cultivar DX and the low-starch cultivar D. The multi-omics data suggest distinct molecular mechanisms underlying differential starch accumulation across tuber tissues, which helps refine the theoretical framework for spatial starch distribution in potato tubers.

Previous physiological studies have observed uneven starch deposition in storage organs of tuberous crops, including cassava, sweet potato, and Chinese yam, with cortical tissues generally showing stronger carbon storage capacity [18]. Nevertheless, the molecular regulatory mechanisms driving such spatial differences remain poorly understood [19]. Our multi-omics data are consistent with these phenotypic observations and further indicate that tissue-level metabolic remodeling may be a key factor contributing to spatial starch heterogeneity in potato tubers.

Transcriptomic profiling reveals widespread gene upregulation in tuber cortex tissues relative to the inner medulla in both DX and D, implying that cortical tissues may establish a metabolically active starch biosynthetic system through extensive transcriptional activation. However, the magnitude of inter-tissue transcriptional variation differs substantially between the two cultivars. The low-starch cultivar D presents 4801 differentially expressed genes (DEGs) between the cortex and the medulla, which is far more than the 2093 DEGs identified in DX. This pattern suggests greater transcriptional fluctuation and potential regulatory disorder across tuber tissues in low-starch germplasm. In contrast, the high-starch cultivar DX displays relatively stable and balanced gene expression among different tuber regions, which may support enhanced inter-tissue metabolic coordination [20]. This regulatory pattern is consistent with previous studies on cereal grains and fleshy fruits, wherein high-efficiency storage germplasm often exhibits stable transcriptional homeostasis and coordinated metabolic networks across tissues [21].

The differentially expressed transcription factors (TFs) identified in this study mainly belong to the AP2/ERF, bHLH, MYB, C2H2, and WRKY families. Accumulated evidence from previous functional studies indicates that AP2/ERF, MYB, and bHLH TFs participate in the regulation of plant carbon partitioning, tuber development, and starch biosynthesis in multiple plant species, including rice, maize, Arabidopsis, and sweet potato [22,23,24]. These TFs can bind to the promoters of starch synthesis-related genes and modulate carbon allocation and storage metabolism [25,26]. Our data imply that such TF-mediated regulatory networks associated with starch metabolism are potentially conserved between dicot tuber crops and monocot cereals.

GO and KEGG enrichment analyses suggest conserved core pathways activated in cortical tissues of both cultivars, including starch and sucrose metabolism, nucleotide sugar metabolism, and sugar transmembrane transport. Coordinated changes in secondary metabolism, hormone signal transduction, and energy homeostasis were also observed, which may constitute a basal regulatory program supporting cortical starch accumulation in potato tubers. Meanwhile, cultivar-specific pathway enrichment patterns were evident. In the low-starch cultivar D, cortical DEGs were preferentially enriched in secondary metabolism, amino acid metabolism, and stress response pathways. Such metabolic reprogramming may divert fixed carbon away from starch biosynthesis and reduce substrate availability for starch polymerization. In comparison, the cortex of the high-starch cultivar DX showed dominant enrichment in carbon biosynthesis and transport pathways, which is consistent with a metabolic pattern that favors carbon allocation to starch production [27]. These observations are in line with previous findings in potato and cassava, indicating that low-starch stress-tolerant germplasm tends to allocate photosynthates to defensive secondary metabolites, whereas high-starch cultivars may reduce unnecessary secondary carbon consumption to prioritize starch synthesis. Studies in Arabidopsis and maize further support a potential competitive relationship between secondary metabolism and starch biosynthesis, which may affect organ carbon storage efficiency.

A total of 1444 metabolites were annotated via untargeted metabolomics. PCA and OPLS-DA results suggest good sample reproducibility and distinct metabolic differences between groups, supporting the reliability of the metabolomic dataset. Differentially expressed metabolites (DMet) exhibited contrasting tissue-specific patterns in the two cultivars: most metabolites decreased in the cortex of cultivar D, while the cortex of DX accumulated numerous upregulated metabolites. This divergent metabolic signature indicates distinct tissue-level metabolic regulatory characteristics between high- and low-starch potato cultivars [28]. KEGG enrichment of DMet highlighted core carbon metabolic pathways, including starch and sucrose metabolism, the TCA cycle, and the pentose phosphate pathway. These metabolomic results are consistent with transcriptomic data, implying that differential carbon metabolic remodeling may underlie spatial starch heterogeneity in potato tubers [29]. The pentose phosphate pathway and the TCA cycle are known to provide energy and reducing power for starch synthesis in tuberous crops, and our findings are consistent with this paradigm and extend the understanding of energy metabolism supporting tuber starch accumulation.

Quantitative analysis of core sugar metabolites suggests obvious tissue metabolic polarization in the low-starch cultivar D. The cortex of D accumulated high levels of sucrose, organic acids, and sugar intermediates, whereas the medulla showed relatively suppressed metabolic activity, forming an imbalanced metabolic phenotype. Despite the abundant sugar substrates in the cortex, enhanced carbon diversion and starch catabolism may limit efficient starch polymerization in D. In contrast, cultivar DX maintained relatively balanced sugar metabolite levels between the cortex and the medulla, with sustained active carbon metabolism across tuber tissues, which may provide stable precursors and energy for continuous starch biosynthesis. These data suggest that the key difference between high- and low-starch cultivars may not simply lie in cortical metabolic activation [30], but rather in the capacity to maintain coordinated whole-tuber carbon metabolism. This finding fills a research gap regarding tissue-specific metabolic differentiation in potato tubers and is consistent with observations in cassava, where high-starch varieties display uniform metabolic activity across storage root tissues while low-starch accessions show severe metabolic polarization.

Integrated transcriptome–metabolome analysis of the starch and sucrose metabolism pathway (ko00500) identified 18 hub structural genes clustered into four functional modules: starch biosynthesis, sucrose transformation, sugar translocation, and starch degradation. In the high-starch cultivar DX, coordinated upregulation of starch synthetic genes (SS16, AGPS1, SPS) and sugar transporters, accompanied by relatively low expression of starch hydrolytic genes (AMYA1, PAIN-1) and the carbon diversion gene TPS, are consistent with an efficient starch accumulation process characterized by sufficient substrate supply, active polymerization, limited degradation, and reduced carbon diversion. In cultivar D, however, this multi-module regulatory network appeared to be dysregulated. Relatively low expression of synthetic genes, together with elevated transcript levels of hydrolytic and carbon diversion genes, may collectively impair starch accumulation. This coordinated regulatory pattern is consistent with reports in high-starch rice, maize, and sweet potato, implying a potential conserved strategy for efficient starch production in high-carbon-storage crops [31], including enhanced biosynthesis, suppressed degradation, limited carbon diversion, and improved substrate transport.

Through comparative multi-omics screening across cultivars and tissues, three conserved hub genes (LOC102593331 TPS, LOC102584887 AMY, and LOC102580651 EN) were identified as candidate negative regulatory genes potentially associated with starch accumulation and spatial starch heterogeneity. These three genes may affect starch metabolism through distinct and synergistic regulatory routes. The EN gene encodes endoglucanase, which participates in cell wall biosynthesis and may compete for UDP-D-glucose substrates, thereby potentially diverting carbon flux away from starch synthesis. Higher EN expression was observed in tuber epidermal tissues of low-starch plants, while its expression was relatively repressed in the medulla of DX, showing a negative correlation with starch accumulation in our omics datasets. The AMY gene encodes α-amylase, which functions in starch hydrolysis; its transcripts are generally enriched in vegetative organs and maintained at low levels in storage tubers, consistent with a potential role in modulating starch turnover. The TPS gene encodes trehalose-6-phosphate synthase [32], a key component of sugar signaling pathways with strong tuber tissue specificity. According to its pathway position, tissue expression pattern, and evolutionary conservation, TPS is proposed as a primary candidate regulatory gene that may modulate global carbon allocation and spatial starch heterogeneity by affecting sugar signal transduction. EN may act as a secondary regulatory gene that potentially limits starch synthesis via substrate competition at terminal metabolic stages, while AMY may serve as an auxiliary factor involved in basal starch metabolic homeostasis.

It is important to note that the carbon flux redistribution proposed in the present study is hypothesized based on transcriptomic and metabolomic correlation data. Further quantitative validation, including metabolic flux measurement, carbon isotope labeling, enzymatic activity assay, and trehalose quantification, is required to confirm the regulatory effects of the candidate genes and carbon pathway reprogramming.

## 5. Conclusions

Transcriptional regulation: Gene upregulation in potato tuber cortical tissues is consistent with enhanced local metabolic activity. The low-starch cultivar D exhibits obvious transcriptional divergence and potential regulatory disorder across tuber tissues, whereas the high-starch cultivar DX maintains relatively balanced and coordinated gene expression between the cortex and the medulla. TF families, including AP2/ERF, bHLH, and MYB, may serve as core upstream regulators potentially mediating tissue-specific starch accumulation patterns.

Metabolic regulation: Cortex and inner medulla tissues display distinct metabolic profiles mainly associated with starch/sucrose metabolism, the TCA cycle, and the pentose phosphate pathway. The low-starch cultivar D shows metabolic polarization, with hyperactive cortical carbon metabolism and suppressed medullary metabolic activity, which may cause inefficient carbon utilization. In comparison, the high-starch cultivar DX presents relatively uniform and balanced carbon metabolism across tuber tissues, which is consistent with sustained substrate supply for starch biosynthesis.

Core genes and pathways: The starch and sucrose metabolic pathway (ko00500) is a key pathway potentially responsible for spatial starch heterogeneity in potato tubers. Eighteen hub structural genes were screened, among which TPS (LOC102593331), AMY (LOC102584887), and EN (LOC102580651) were identified as candidate conserved negative regulatory genes. Transcriptomic and metabolomic correlation data suggest that these three genes may inhibit starch accumulation through independent pathways, including potential carbon flux diversion, starch hydrolytic degradation, and cell wall substrate competition.

In summary, the high-starch cultivar DX exhibits coordinated whole-tuber carbon metabolism, which is consistent with activated starch biosynthesis and transport and repressed starch degradation and carbon diversion. In contrast, the low-starch cultivar D displays dysregulated metabolic networks, imbalanced carbon partitioning, and tissue metabolic polarization, which may restrict starch accumulation. Combined with our previous results, the present study provides multi-omics evidence to refine the theoretical framework for carbon allocation during potato starch accumulation and offers valuable candidate gene resources for molecular breeding of high-starch potato varieties.

## Data Availability

All data generated or analyzed during this study are included in this published article and its Supplementary Information Files.

## Supplementary Materials

The following supporting information can be downloaded at https://www.mdpi.com/article/10.3390/biology15181655/s1, Table S1. Primers for quantitative real-time PCR (qRT-PCR); Table S2. Starch content (%) in three regions of DX at different stages; Table S3. Starch content (%) in three regions of D at different stages; Table S4. Summary of RNA-seq sequencing quality and genome mapping statistics for all samples; Table S5. FPKM expression matrix of candidate genes in tubers of two potato varieties at different developmental stages; Table S6. Summary of differentially expressed genes in D; Table S7. Summary of differentially expressed genes in DX; Table S8. GO functional enrichment results of differentially expressed genes (Biological Process); Table S9. GO Biological Process enrichment of differentially expressed genes in potato (dominated by carbohydrate metabolism); Table S10. Gene expression levels of two potato cultivars (D and DX) at two developmental stages with biological replicates; Table S11. KEGG pathway enrichment statistics of differentially expressed genes in D and DX; Table S12. Metabolite contents of two potato cultivars (D and DX) at two developmental stages; Table S13. Correlation analysis between key starch synthesis genes (SS16, AGPS1) and carbohydrate metabolites in potato; Table S14. Correlation analysis table of metabolites and gene expression levels in potato tubers; Table S15. RT-PCR validation of RNA-seq gene expression; Table S16. Gene expression levels of genes in different tissues of DX; Table S17. Results of two_x001e_way repeated-measures ANOVA for starch content in tubers of cultivar DX; Table S18. Results of two_x001e_way repeated-measures ANOVA for starch content in tubers of cultivar D.
